# Cancer Stem Cell Gene Profile as Predictor of Relapse in High Risk Stage II and Stage III, Radically Resected Colon Cancer Patients

**DOI:** 10.1371/journal.pone.0072843

**Published:** 2013-09-04

**Authors:** Riccardo Giampieri, Mario Scartozzi, Cristian Loretelli, Francesco Piva, Alessandra Mandolesi, Giovanni Lezoche, Michela Del Prete, Alessandro Bittoni, Luca Faloppi, Maristella Bianconi, Luca Cecchini, Mario Guerrieri, Italo Bearzi, Stefano Cascinu

**Affiliations:** 1 Department of Medical Oncology, AO Ospedali Riuniti-UNIVPM, Ancona, Italy; 2 UNIVPM, Ancona, Italy; 3 Institute of Pathology, AO ospedali Riuniti-UNIVPM, Ancona, Italy; 4 Institute of Surgery, AO ospedali Riuniti-UNIVPM, Ancona, Italy; University of Navarra, Spain

## Abstract

Clinical data indicate that prognostic stratification of radically resected colorectal cancer based on disease stage only may not be always be adequate. Preclinical findings suggest that cancer stem cells may influence the biological behaviour of colorectal cancer independently from stage: objective of the study was to assess whether a panel of stemness markers were correlated with clinical outcome in resected stage II and III colon cancer patients. A panel of 66 markers of stemness were analysed and thus patients were divided into two groups (A and B) with most patients clustering in a manner consistent with different time to relapse by using a statistical algorithm. A total of 62 patients were analysed. Thirty-six (58%) relapsed during the follow-up period (range 1.63–86.5 months). Twelve (19%) and 50 (81%) patients were allocated into group A and B, respectively. A significantly different median relapse-free survival was observed between the 2 groups (22.18 vs 42.85 months, p = 0.0296). Among of all genes tested, those with the higher “weight” in determining different prognosis were CD44, ALCAM, DTX2, HSPA9, CCNA2, PDX1, MYST1, COL1A1 and ABCG2. This analysis supports the idea that, other than stage, biological variables, such as expression levels of colon cancer stem cell genes, may be relevant in determining an increased risk of relapse in resected colorectal cancer patients.

## Introduction

Surgery remains the mainstay of treatment of non-metastatic colorectal cancer patients and in about 50% stage III resected patients, cure is achieved by surgery alone. Adjuvant treatment trials such as the MOSAIC [1] or XELOXA trial [2] showed that combination chemotherapy with 5FU/Capecitabine and Oxaliplatin yields a survival advantage in the ranges of 10–15% for stage III resected patients.

In stage II patients, data are even more limited: adjuvant chemotherapy is usually prescribed on the basis of risk factors or “the oncologist’s choice”: the proportion of stage II patients enrolled in clinical trials is too small to make definitive assumptions regarding the benefit of adjuvant treatment in this subset of patients [3]–[5].

To date the TNM staging system cathegories are the most reliable way to address patients’ risk to relapse for decisions about clinical management. A possible role for cancer stem cells has been suggested as potential predictor of high risk of relapse in resected colorectal cancer patients. These cells have been identified as predictors of poor outcome and implied to have a role in cancer progression and development of metastases, possibly as a consequence of their hypothetical high replication potential [6], [7].

Canonically, colorectal cancer stem cell population may be primarily divided into 2 main classes: a typical subtype of colorectal cancer stem cells, usually identified by CD133 positive stain and a heterogeneous population of non-CD133 positive stem cells. However both classes seem to possess an equally effective capability to proliferate under certain conditions [8]–[10]. Due to their biological characteristics cancer stem cells represent an almost limitless resource for tumour growth and progression and are thought to be responsible for maintaining a sort of protected reserve niche for cancer cells. A further peculiarity of cancer stem cells is their relative ability to escape chemotherapy-induced cell death, thus reinforcing their role as a self-renewing cancer cells source for tumours [11]–[13].

Based on these assumptions we can hypothesise that the cancer stem cell population may be responsible for the biological characteristics of tumour cells and may ultimately influence clinical behaviour of solid tumours even during chemotherapy treatment.

In a previous analysis of Gerger et al. [14] the Authors were able to identify common cancer stem cell gene variants as predictive factors for recurrence in radically resected colon cancer patients. In this study germline polymorphisms of a limited number of putative stem cells markers have been analysed. However it is possible that stem cell gene profile in tumours may differ from what can be observed at the germline level. Moreover stem cell gene expression in tumours may also be biologically relevant.

In our study we assessed whether a panel of 66 genes, indicated to have a role in growth and proliferation of colon cancer stem cells, could allow to make a more accurate prediction of the likelihood of relapse in resected non-metastatic colon cancer patients. The final aim was to identify a stemness-based risk category and to indicate possible stem cells-linked molecular targets for future development of stem cell-directed treatment strategies.

## Materials and Methods

### Patients Selection

Radically resected colon cancer patients receiving adjuvant chemotherapy at our Department from 2005 to 2007 were considered eligible for our study.

Patients should have presented either with a clinically defined high risk stage II or with a stage III completely resected colon tumour.

High risk stage II was defined in presence of at least one of the following: pT4, poorly differentiated histology, bowel obstruction or perforation at presentation, histologically proven vascular, lymphatic or perineural invasion. We excluded from analysis patients receiving adjuvant chemotherapy exclusively on the basis of a non adequate lymph node sampling (lymph node sampling less than 12).

Either single agent 5FU (or capecitabine) or 5FU (or capecitabine) in combination with oxaliplatin were considered as acceptable alternatives. Follow-up occurred according to Institutional guidelines. History, physical examination, a complete blood count, CEA and CA 19-9 determination were performed at three-months intervals for three years, then at six months intervals at years 4 and 5 after surgery. CT scan of chest and abdomen was done every 6 months at year 1 through 3 and yearly thereafter at years 4 and 5. Colonoscopy was performed at year 1 and then every 3 and 5 years. The site and date of first relapse and the date of death were recorded. For study purposes patients data were retrospectively analysed. This study was approved by our Institutional Ethical Committee (Comitato Etico Azienda Ospedaliera - Ospedali Riuniti, Ancona, Via Conca 71, Italy). All patients gave their written consent to the study.

### Samples Processing and Quantitative PCR Analysis

Gene expression profile analysis was performed by laboratory personnel blinded to patients’ status.

Multiple sections of formalin-fixed, paraffin-embedded tissue blocks (25 to 30 mg of primary tumour, manual microdissected tissue) were collected; paraffin wax was removed and total RNA was extracted by the RT^2^ FFPE RNA Extraction Kit (SABiosciences Corporation, Frederick, MD, USA), following the manufacturer’s instructions. RNA samples were quantified and quality-tested for the presence of protein and/or organic solvent contaminants by a spectrophotometric assay.

Five hundred nanograms from each sample were reverse transcribed to cDNA and pre-amplified using the RT2 FFPE PreAMP cDNA Synthesis Kit and the primer mix specific for the customized Stem Cell RT2Profiler PCR Array (SABiosciences Corporation).

Quantitative Real Time PCR analysis was performed on a 7300 Real-Time PCR System (Applied Biosystems Inc, Foster City, CA, USA) by a SYBR® Green method. Target and reference genes and controls selected for gene expression analysis came from the Stem Cell genes set of the Stem Cells RT2Profiler PCR Array (#CAPH09495-PAHS-405, SABiosciences Corporation). Also, due to the lack in the standard PCR array of Stem Cell gene markers more proper to colorectal cancer stem cells, the specific genes ALCAM (also known as CD166), PROM1 (CD133), CD24 and LGR5 were added.

A complete list of the genes tested can be found in Table 1.

**Table 1:** Genes analysed and main gene function.

Stemness Markers						
Cell Cycle Regulators	APC	AXIN1	CCNA2	CCND1	CCND2	CCNE1
	CDC2	CDC42	EP300	FGF1	FGF2	FGF3
	FGF4	MYC	NOTCH2	PARD6A	RB1	
Chromosome and Chromatin Modulators	GCN5L2	HDAC2	MYST1	MYST2	RB1	TERT.
Cell Division	DHH	NOTCH1	NOTCH2	NUMB	PARD6A	
Self-Renewal Markers	HSPA9	MYST1	MYST2	NEUROG2	SOX1	SOX2
Cytokines and Growth Factors	BMP1	BMP2	BMP3	CXCL12	FGF1	FGF2
	FGF3	FGF4	GDF2	GDF3	IGF1	JAG1
Cell-Cell Communication	DHH	DLL1	GJA1	GJB1	GJB2	JAG1.
Cell Adhesion Molecules	APC	BGLAP	CD4	CD44	CDH1	CDH2
	COL9A1	CTNNA1	CXCL12	NCAM1		
Metabolic Markers	ABCG2	ALDH1A1	ALDH2	FGFR1		
**Stem Cell Differentiation Markers**						
Embryonic Cell Lineage Markers	ACTC1	ASCL2	FOXA2	PDX1	ISL1	KRT15
	MSX1	MYOD1	T			
Hematopoietic Cell Lineage Markers	CD3D	CD4	CD8A	CD8B	MME	
Mesenchymal Cell Lineage Markers	ACAN	ALPI	BGLAP	COL1A1	COL2A1	COL9A1
	PPARG					
Neural Cell Lineage Markers	CD44	NCAM1	OPRS1	S100B	TUBB3	
**Stem Cell Maintenance Pathways**						
Notch Pathway	DLL1	DLL3	DTX1	DTX2	DVL1	EP300
	GCN5L2	HDAC2	JAG1	NOTCH1	NOTCH2	NUMB
Wnt Pathway	ADAR	APC	AXIN1	BTRC	CCND1	FRAT1
	FZD1	MYC	PPARD	WNT1		
**Cancer Stem Cells Markers**	ALCAM	CD133	CD44	LGR5	SOX2	ALDH1A1

### Data Processing and Statistical Analysis

Relative gene expression was quantified using the comparative ΔCt method. We used the tool “PCR Array Data Analysis Web Portal” (http://www.SABiosciences.com/pcrarraydataanalysis.php) on the manufacturer’s website to perform data quality tests, calculations on the qPCR data and data normalization. In particular, all threshold cycles values greater than 35, or not detected, were considered as negative calls. We retained samples with negative genomic DNA control, definite reverse transcription control and positive PCR control values, according to manufacturer’s indications.

To evaluate different patterns of gene expression, we conducted clustering analysis by K-means (K = 2) method [15]. This particular clustering analysis algorithm was chosen to test the possible existence of 2 different patterns of gene expression, related to different prognosis. In particular, the K-means method aims to form pre-defined number (K) partitions into a cluster of different observations in a data set, by the method of the nearest mean. Clustering analyses were performed by MEV (MultiExperiment Viewer) tool.

### Statistical Analysis was Performed with MedCalc Package (MedCalc® v9.4.2.0)

Primary endpoint of the study was to identify between the 2 prognostic groups a significant difference in median time to relapse (TTR), calculated as the interval from the date of diagnosis to the date of tumour recurrence. TTR was censored at the time of death or last follow up if the patient had not recurred.

In the primary hypothesis that patients with resected high-risk stage II-stage III colorectal cancer have around 60% relapse-free risk at 2-years observation time and that patients with poor prognostic score have 30% relapse-free risk at 2-years, assuming alpha-probability error of 0.05 and beta-probability error of 0.10, a minimum of 53 patients are needed to test the hypothesis.

The association between TTR and cancer stem cell genetic profile was estimated by the Kaplan-Meier method. Significant differences in probability of relapsing between the strata were evaluated by log-rank test.

For each gene identified by K-means clustering analysis it was subsequently evaluated the correlation with time to relapse: the median of the overall specific gene concentration was used as cut-off, due to lack of other more reliable and reproducible means to estabilish a proper cut-off.

## Results

A total of 62 patients were analysed. Among them, 36 had a stage II colon cancer (58%) whereas the remaining 26 (41%) had a stage III disease (table 2). Median follow-up period was 44 months (range 12.5–86.5 months). During this follow-up period 36 (58%) patients relapsed.

**Table 2:** Patients characteristics.

	Whole Group (n = 62)	Group A	Group B	P value
**Age (range)**	64 (36–80)	65 (38–78)	64 (36–80)	0.95
**Sex**				
Males	41 (66%)	8 (66%)	33 (66%)	0.76
Females	21 (37%)	4 (34%)	17 (34%)	
**Stage II**	**36 (58%)**	**10 (83%)**	**26 (52%)**	**0.09**
- pT4a	11 (30%)	3 (25%)	8 (31%)	0.75
- Obstruction/Perforation	8 (22%)	3 (25%)	5 (19%)	0.36
- Vascular/Lymphatic/Perineural invasion	10 (28%)	4 (33%)	6 (23%)	0.17
- Poorly differentiated	13 (36%)	4 (33%)	10 (38%)	0.54
**Stage III**	**26 (42%)**	**2 (17%)**	**24 (48%)**	**0.09**
-pN1	20 (77%)	2 (100%)	18 (75%)	0.94
-pN2	6 (23%)	0 (0%)	6 (25%)	
**Treatment**				
- Fluoropyrimidines	28 (45%)	4 (33%)	17 (34%)	0.76
- Oxaliplatin combinations	34 (55%)	8 (67%)	33 (66%)	

Among 26 stage III patients, 10 (38%) relapsed during the follow-up period whereas in the remaining 36 stage II patients, 26 (72%) relapses were seen.

Median TTR for stage II patients was 23.3 months, whereas median TTR was not reached for stage III patients. This difference was not statistically significant (p = 0.0696).

**Figure 1:**
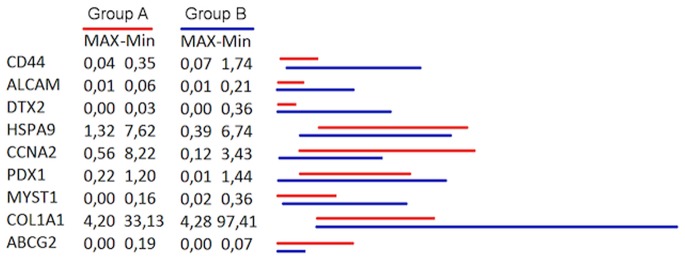
Comparison of gene expression means between group A and B. Different gene expression means between patients with radically resected colon cancer patients showing an unfavourable cancer stem cell gene profile, GROUP A, (red) and radically resected colon cancer patients showing a favourable cancer stem cell gene profile, GROUP B (blue) as stratified by K-means (K = 2) method.

When performing K-means analysis (K = 2), genes that had a major role in determining allocation into one of the 2 pre-determined groups were CD44 (p = 0.0004), ALCAM (p = 0.003), DTX (p = 0.005), HSPA9 (p = 0.012), CCNA (p = 0.03), PDX1 (p = 0.04), MYST1 (p = 0.04), COL1A1 (p = 0.03), ABCG2 (p = 0.04). A comparison of the different means of expression of these genes in group A and B can be found in Figure 1.

After clustering analysis two groups of patients were identified: group A (unfavourable cancer stem cell gene profile) and group B (favourable cancer stem cell gene profile).

Twelve patients (19%) were allocated by K-means analysis into group A, whereas the remaining 50 patients (81%) were allocated into group B. In group A, 2 stage III (17%) and 10 stage II (83%) patients were allocated, whereas in group B the remaining 24 (48%) stage III and 26 (52%) stage II patients were allocated. All the others clinical characteristics analysed resulted well balanced between the 2 groups (table 2).

**Figure 2:**
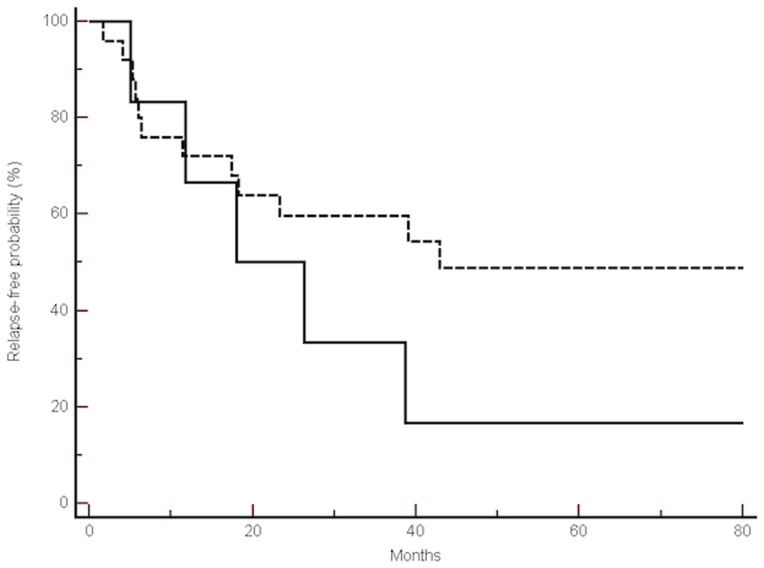
Comparison of median time to relapse between group A and B. Kaplan-Meier curves for median time to relapse (TTR) of radically resected colon cancer patients showing an unfavourable cancer stem cell gene profile, GROUP A (——) vs. radically resected colon cancer patients showing a favourable cancer stem cell gene profile, GROUP B (-------) (22.1 months vs. 42.8 months, p = 0.02).

Eleven (91%) patients in group A relapsed during the observation period whereas 25 (50%) patients in group B relapsed during the same period. This difference was statistically significant at the chi-square test (p = 0.0214). A significantly different median TTR between the 2 groups was observed: 22.18 vs 42.85 months (HR: 0.46, 95%CI: 0.15–090, p = 0.02) (Figure 2).

When analysing the impact of every single gene identified by K-means analysis taken singularly, no significant relationship with median TTR was observed. A summary of these results can be found in Table 3.

**Table 3:** Impact of the genes identified by K-means analysis and CD133 when analysed singularly and relapse risk and time-to-relapse.

Gene	Relapse free survival(Hi vs Low)	HR (95%CI)	p
**CD44**	33.24 vs 42.85	0.73 (0.29–1.86)	0.52
**ALCAM**	28.91 vs 42.00	0.59 (0.30–1.10)	0.09
**DTX2**	33.24 vs 42.03	0.90 (0.46–1.76)	0.76
**HSPA9**	39.01 vs 42.03	0.98 (0.50–1.90)	0.95
**CCNA2**	36.25 vs 42.03	0.72 (0.37–1.40)	0.33
**PDX1**	37.63 vs NR	0.68 (0.34–1.31)	0.25
**MYST1**	42.03 vs 39.01	0.78 (0.40–1.51)	0.46
**COL1A1**	39.01 vs NR	0.63 (0.32–1.22)	0.17
**ABCG2**	36.12 vs NR	0.31 (0.36–1.38)	0.71
**CD133**	42.85 vs 39.01	1.26 (0.50–3.23)	0.61

## Discussion

Identifying a subset of radically resected, non-metastatic colorectal cancer patients who are at higher likelihood to relapse has deep implications. TNM staging system, even if accounting for tumour diffusion as the main factor influencing risk of relapse does not account for biological characteristics of the tumour itself.

A possible role for cancer stem cells has been hypothesized as potential predictor of high risk of relapse in resected colon cancer patients [6]–[13]. These cells have been usually identified as predictors of poor outcome and implied to have a role in cancer progression and development of metastases. In our analysis we suggested that cancer stem cell gene profile may be relevant in determining TTR in high-risk stage II and stage III radically resected colon tumours. Moreover the risk of relapse in our series seemed not correlated with disease stage, suggesting that biology more than stage guides natural history of colon cancer. When performing K-means analysis (K = 2), genes who had a major role in determining allocation into one of the 2 pre-determined groups were CD44 (p = 0.0004), ALCAM (p = 0.003), DTX (p = 0.005), HSPA9 (p = 0.012), CCNA (p = 0.03), PDX1 (p = 0.04), MYST1 (p = 0.04), COL1A1 (p = 0.03), ABCG2 (p = 0.04).

A role for CD44, ALCAM, DTX, HSPA9, CCNA, PDX1, MYST1, COL1A1 and ABCG2 expression in influencing tumour outcome, possibly through interaction with the stem cells population, has been separately described in the past, but this is, to our knowledge, the first time that multiple markers have been examined simultaneously, confirming the relative significance of each one of them.

However, among the molecular determinants emerged as significant in our study, we believe that results regarding CD44, ABCG2 and CD133 should be discussed further, especially because of the biological peculiarity they possess.

In particular CD44 gene expression seemed to represent the most important factor influencing the likelihood of relapse in our group of resected patients. CD44 is also known as the receptor for hyaluronic acid and has an important role in cells to stroma interaction [16]–[19]. Although already reported as a predictive factor for recurrence in colorectal cancer patients, substantial controversy exists about the actual impact of different levels of CD44 gene expression. In a previous work by Huang et al. [20] stem cells taken from colorectal cancer patients were evaluated for gene expression of CD44 and CD133 and tumours harbouring higher levels of both CD44 and CD133 were related to higher risk of development of early liver metastases.

On the contrary, in a work published by Dallas et al. [21] cells that were engineered to be knock-down for CD44 expression had almost 10 fold increase in metastatic potential in both liver and lung. In addition to that, CD44 negative cells exhibited a greater “mechanical compliance” (the capacity for cytosol components to move freely through the cytosol itself), a property that is considered crucial in the process of extravasation and migration through the blood-stream. Globally these findings seem to go along with our observation that low levels of CD44 are relevant for time to relapse in colon cancer.

On the contrary higher levels of expression of ABCG2 resulted more frequently present in group A patients, those with worse prognosis. ABCG2 (also known as CDw338) is a protein on the cell surface, involved with transport of molecules across cell membranes. Expression of this protein has been related with multi-drug resistance and in a recent work of Oh et al. [22] colorectal cancer cells becoming resistant to FOLFOX chemotherapy exhibited high levels of ABCG2. This could explain the apparent reduced efficacy of adjuvant chemotherapy in our group of patients with worse prognosis.

Even though published data so far seem to suggest that CD133 represents an important biomarker in colon cancer stem cells, its role is far from being fully understood. This protein (also called Prominin-1 or in abbreviated form PROM-1) has a role in the formation of membrane protrusions and vescicular trafficking [23]. Other than this rather base function, it is postulated that PROM-1 interacts with other well-and-not-so-well known intracellular messangers such as those involved in WNT/Beta-catenin, PI3K-Akt-mTOR, HIF-1alfa and CXCR4 pathways [24], [25].

In particular, high CD133 expression seems to be related to worse prognosis in colorectal cancer due to its higher incidence in metastases rather than in primary tumour [17].

In our analysis, different levels of CD133 were not linked to a higher likelihood of relapse. Indeed, patients in group A and B had heterogeneous levels of CD133 expression, with no significant differences among the two groups. Also, when analysing the impact of high vs low concentration of CD133 no significant difference with TTR was observed.

These data seem to be in contrast with those presented by Artells et al. [26] suggesting that high levels of CD133 gene expression were correlated with a higher likelihood of relapse in resected colorectal cancer patients. Our results may be explained by the fact that in our analysis several different factors were taken into account and not just one marker of stemness: due to the nature of K-means analysis, in presence of multiple biological factors with a more definite impact on TTR, the effect of CD133 as marker of relapse could be diluted.

We also know that CD133 IHC expression may be inducible and that even previously CD133 null stem cells may express *de novo* CD133 in different culture means and under specific conditions [27]. This could at least account for the contradicting results of analysis conducted on CD133 IHC expression and prognosis.

Furthermore, in another experience of Shmelkov et al. [8] CD133 null cells were hypothesised to possess even a more aggressive phenotype than their counterpart CD133 positive. In particular, when inoculated into SCIV mices, tumor growth from CD133 negative stem cells was markedly greater than CD133 positive cell population.

Globally our findings suggest for the first time a potential role for cancer stem cell gene profile in discriminating different risks of relapse irrespectively of disease stage. These results may be also relevant, after further confirmation, for the identification of possible stem cells-linked molecular targets for future development of stem cell-directed treatment strategies.
